# Supplementary material for: The Risk for Neonatal Hypoglycemia and Bradycardia after Beta-Blocker Use during Pregnancy or Lactation: A Systematic Review and Meta-Analysis
Source: Int J Environ Res Public Health. 2022 Aug 4;19(15):9616. doi: 10.3390/ijerph19159616 (PMC9368631; doi:10.3390/ijerph19159616)
Supplement: Supplementary file 1 [file ijerph-19-09616-s001.zip › ijerph-1817843-supplementary.pdf]

Supplemental file: Search strategy

embase.com	865	851
Medline ALL Ovid	322	94
Web of Science SCI-EXPANDED & SSCI	187	69
Cochrane CENTRAL register of trials	88	29
**Total**	**1462**	**1043**

embase.com

('beta adrenergic receptor blocking agent'/exp OR (((beta* OR β OR β1 OR β2 OR β3) NEAR/3 (block* OR antagonist*)) OR adaprolol* OR afurolol* OR alprenolol* OR befunolol* OR bfe-55* OR bopindolol* OR bornaprolol* OR bromoacetylalprenololmenthane* OR bucindolol* OR bucumolol* OR bufetolol* OR bufuralol* OR bunitrolol* OR bunolol* OR bupranolol* OR butofilolol* OR carazolol* OR carpindolol* OR carteolol* OR carvedilol* OR cloranolol* OR deacetylmetipranolol* OR dexpropranolol* OR diacetolol* OR dichlorisoprenaline* OR dihydroalprenolol* OR dilevalol* OR diprafenone* OR ersentilide* OR exaprolol* OR falintolol* OR flestolol* OR hydroxybenzylpindolol* OR indenolol* OR iodopindolol* OR iprocrolol* OR isamoltane* OR isoxaprolol* OR labetalol* OR levobunolol* OR levomoprolol* OR mepindolol* OR mercuderamide* OR metipranolol* OR moprolol* OR nadolol* OR nifenalol* OR oberadilol* OR oxprenolol* OR pafenolol* OR pamatolol* OR penbutolol* OR pindolol* OR primidolol* OR prizidilol* OR procinolol* OR pronetalol* OR propranolol* OR proxodolol* OR ridazolol* OR soquinolol* OR sotalol* OR spirendolol* OR tazolol* OR tertatolol* OR tienoxolol* OR tilisolol* OR timolol* OR tolamolol* OR toliprolol* OR trasitensin* OR trepress* OR tribendilol* OR viskaldix* OR xibenolol* OR zoleprodolol* OR acebutolol* OR alpha-hydroxymetoprolol* OR atenolol* OR bendacalol* OR betaxolol* OR bevantolol* OR bisoprolol* OR celiprolol* OR cetamolol* OR cyanoiodopindolol* OR cyanopindolol* OR dramedilol* OR epanolol* OR esmolol* OR flusoxolol* OR landiolol* OR metoprolol* OR nebivolol* OR practolol* OR ritodrine* OR salcardolol* OR sandoz-204545* OR talinolol* OR vortioxetine* OR bendacalol* OR butoxamine* OR cicloprolol* OR arotinolol*):Ab,ti) AND (pregnancy/exp OR 'pregnant woman'/de OR 'prenatal exposure'/de OR 'breast feeding'/exp OR 'breast milk'/de OR lactation/de OR 'maternal exposure'/de OR 'maternal treatment'/de OR 'prenatal drug exposure'/de OR 'prenatal period'/de OR 'maternal hypertension'/de OR 'pregnancy disorder'/de OR 'pregnancy outcome'/exp OR 'drug milk level'/de OR 'placental transfer'/de OR 'perinatal drug exposure'/de OR (pregnan* OR prenatal* OR (breast NEXT/1 (feed* OR fed OR milk)) OR human-milk OR lactat* OR intra-uterin* OR intrauterin* OR ((fetus* OR foetus* OR fetal* OR foetal*) NEAR/3 expos*) OR mother OR maternal OR placenta* OR perinatal* OR peri-natal*):Ab,ti) AND (bradycardia/exp OR 'adverse event'/de OR 'adverse drug reaction'/de OR 'side effect'/de OR hypoglycemia/de OR hypoglycemia/de OR 'toxicity and intoxication'/exp OR complication/de OR (adverse* OR side-effect* OR bradycard* OR hypoglycemi* OR hypoglycaemia OR harm* OR toxic* OR intoxic* OR complication*):ab,ti) AND (newborn/exp OR (newborn* OR new-born* OR neonat* OR postnatal*):ab,ti) NOT ([animals]/lim NOT [humans]/lim) NOT ([conference abstract]/lim AND [2000-2018]/py) AND [english]/lim

Medline ALL Ovid 640

(exp Adrenergic beta-Antagonists/ OR (((beta*) ADJ3 (block* OR antagonist*)) OR adaprolol* OR afurolol* OR alprenolol* OR befunolol* OR bfe-55* OR bopindolol* OR bornaprolol* OR bromoacetylalprenololmenthane* OR bucindolol* OR bucumolol* OR bufetolol* OR bufuralol* OR bunitrolol* OR bunolol* OR bupranolol* OR butofilolol* OR carazolol* OR carpindolol* OR carteolol* OR carvedilol* OR cloranolol* OR deacetylmetipranolol* OR dexpropranolol* OR diacetolol* OR dichlorisoprenaline* OR dihydroalprenolol* OR dilevalol* OR diprafenone* OR ersentilide* OR exaprolol* OR falintolol* OR flestolol* OR hydroxybenzylpindolol* OR indenolol* OR iodopindolol* OR iprocrolol* OR isamoltane* OR isoxaprolol* OR labetalol* OR levobunolol* OR levomoprolol* OR mepindolol* OR mercuderamide* OR metipranolol* OR moprolol* OR nadolol* OR nifenalol* OR oberadilol* OR oxprenolol* OR pafenolol* OR pamatolol* OR penbutolol* OR pindolol* OR primidolol* OR prizidilol* OR procinolol* OR pronetalol* OR propranolol* OR proxodolol* OR ridazolol* OR soquinolol* OR sotalol* OR spirendolol* OR tazolol* OR tertatolol* OR tienoxolol* OR tilisolol* OR timolol* OR tolamolol* OR toliprolol* OR trasitensin* OR trepress* OR tribendilol* OR viskaldix* OR xibenolol* OR zoleprodolol* OR acebutolol* OR alpha-hydroxymetoprolol* OR atenolol* OR bendacalol* OR betaxolol* OR bevantolol* OR bisoprolol* OR celiprolol* OR cetamolol* OR cyanoiodopindolol* OR cyanopindolol* OR dramedilol* OR epanolol* OR esmolol* OR flusoxolol* OR landiolol* OR metoprolol* OR nebivolol* OR practolol* OR ritodrine* OR salcardolol* OR sandoz-204545* OR talinolol* OR vortioxetine* OR bendacalol* OR butoxamine* OR cicloprolol* OR arotinolol*).ab,ti.) AND (exp Pregnancy/ OR Pregnant Women/ OR Breast Feeding/ OR Milk, Human/ OR Lactation/ OR Maternal Exposure/ OR Maternal-Fetal Exchange/ OR Prenatal Exposure Delayed Effects/ OR Fetal Growth Retardation/ OR exp Pregnancy Outcome/ OR (pregnan* OR prenatal* OR (breast ADJ (feed* OR fed OR milk)) OR human-milk OR lactat* OR intra-uterin* OR intrauterin* OR ((fetus* OR foetus* OR fetal* OR foetal*) ADJ3 expos*) OR mother OR maternal OR placenta* OR perinatal* OR peri-natal*).ab,ti.) AND (Bradycardia / OR Drug-Related Side Effects and Adverse Reactions / OR side effect/ OR Hypoglycemia / OR toxicity.xs. OR complication.xs. OR (adverse* OR side-effect* OR bradycard* OR hypoglycemi* OR hypoglycaemia OR harm* OR toxic* OR intoxic* OR complication*).ab,ti.) AND (Infant, Newborn / OR (newborn* OR new-born* OR neonat* OR postnatal*).ab,ti.) NOT (exp animals/ NOT humans/) AND english.la.

Web of Science SCI-EXPANDED & SSCI

TS=(((((beta* OR β OR β1 OR β2 OR β3) NEAR/2 (block* OR antagonist*)) OR adaprolol* OR afurolol* OR alprenolol* OR befunolol* OR bfe-55* OR bopindolol* OR bornaprolol* OR bromoacetylalprenololmenthane* OR bucindolol* OR bucumolol* OR bufetolol* OR bufuralol* OR bunitrolol* OR bunolol* OR bupranolol* OR butofilolol* OR carazolol* OR carpindolol* OR carteolol* OR carvedilol* OR cloranolol* OR deacetylmetipranolol* OR dexpropranolol* OR diacetolol* OR dichlorisoprenaline* OR dihydroalprenolol* OR dilevalol* OR diprafenone* OR ersentilide* OR exaprolol* OR falintolol* OR flestolol* OR hydroxybenzylpindolol* OR indenolol* OR iodopindolol* OR iprocrolol* OR isamoltane* OR isoxaprolol* OR labetalol* OR levobunolol* OR levomoprolol* OR mepindolol* OR mercuderamide* OR metipranolol* OR moprolol* OR nadolol* OR nifenalol* OR oberadilol* OR oxprenolol* OR pafenolol* OR pamatolol* OR penbutolol* OR pindolol* OR primidolol* OR prizidilol* OR procinolol* OR pronetalol* OR propranolol* OR proxodolol* OR ridazolol* OR

soquinolol* OR sotalol* OR spirendolol* OR tazolol* OR tertatolol* OR tienoxolol* OR tilisolol* OR timolol* OR tolamolol* OR toliprolol* OR trasitensin* OR trepress* OR tribendilol* OR viskaldix* OR xibenolol* OR zoleprodolol* OR acebutolol* OR alpha-hydroxymetoprolol* OR atenolol* OR bendacalol* OR betaxolol* OR bevantolol* OR bisoprolol* OR celiprolol* OR cetamolol* OR cyanoiodopindolol* OR cyanopindolol* OR dramedilol* OR epanolol* OR esmolol* OR flusoxolol* OR landiolol* OR metoprolol* OR nebivolol* OR practolol* OR ritodrine* OR salcardolol* OR sandoz-204545* OR talinolol* OR vortioxetine* OR bendacalol* OR butoxamine* OR cicloprolol* OR arotinolol*)) AND ((pregnan* OR prenatal* OR (breast NEAR/1 (feed* OR fed OR milk)) OR human-milk OR lactat* OR intra-uterin* OR intrauterin* OR ((fetus* OR foetus* OR fetal* OR foetal*) NEAR/2 expos*) OR mother OR maternal OR placenta* OR perinatal* OR peri-natal*)) AND ((adverse* OR side-effect* OR bradycard* OR hypoglycemi* OR hypoglycaemia OR harm* OR toxic* OR intoxic* OR complication*)) AND ((newborn* OR new-born* OR neonat* OR postnatal*))) AND DT=(article) AND LA=(english)

Cochrane CENTRAL register of trials

((((beta* OR β OR β1 OR β2 OR β3) NEAR/3 (block* OR antagonist*)) OR adaprolol* OR afurolol* OR alprenolol* OR befunolol* OR bfe NEXT 55* OR bopindolol* OR bornaprolol* OR bromoacetylalprenololmenthane* OR bucindolol* OR bucumolol* OR bufetolol* OR bufuralol* OR bunitrolol* OR bunolol* OR bupranolol* OR butofilolol* OR carazolol* OR carpindolol* OR carteolol* OR carvedilol* OR cloranolol* OR deacetylmetipranolol* OR dexpropranolol* OR diacetolol* OR dichlorisoprenaline* OR dihydroalprenolol* OR dilevalol* OR diprafenone* OR ersentilide* OR exaprolol* OR falintolol* OR flestolol* OR hydroxybenzylpindolol* OR indenolol* OR iodopindolol* OR iprocrolol* OR isamoltane* OR isoxaprolol* OR labetalol* OR levobunolol* OR levomoprolol* OR mepindolol* OR mercuderamide* OR metipranolol* OR moprolol* OR nadolol* OR nifenalol* OR oberadilol* OR oxprenolol* OR pafenolol* OR pamatolol* OR penbutolol* OR pindolol* OR primidolol* OR prizidilol* OR procinolol* OR pronetalol* OR propranolol* OR proxodolol* OR ridazolol* OR soquinolol* OR sotalol* OR spirendolol* OR tazolol* OR tertatolol* OR tienoxolol* OR tilisolol* OR timolol* OR tolamolol* OR toliprolol* OR trasitensin* OR trepress* OR tribendilol* OR viskaldix* OR xibenolol* OR zoleprodolol* OR acebutolol* OR alpha NEXT hydroxymetoprolol* OR atenolol* OR bendacalol* OR betaxolol* OR bevantolol* OR bisoprolol* OR celiprolol* OR cetamolol* OR cyanoiodopindolol* OR cyanopindolol* OR dramedilol* OR epanolol* OR esmolol* OR flusoxolol* OR landiolol* OR metoprolol* OR nebivolol* OR practolol* OR ritodrine* OR salcardolol* OR sandoz NEXT 204545* OR talinolol* OR vortioxetine* OR bendacalol* OR butoxamine* OR cicloprolol* OR arotinolol*):Ab,ti) AND ((pregnan* OR prenatal* OR (breast NEXT/1 (feed* OR fed OR milk)) OR human NEXT milk OR lactat* OR intra NEXT uterin* OR intrauterin* OR ((fetus* OR foetus* OR fetal* OR foetal*) NEAR/3 expos*) OR mother OR maternal OR placenta* OR perinatal* OR peri NEXT natal*):Ab,ti) AND ((adverse* OR side NEXT effect* OR bradycard* OR hypoglycemi* OR hypoglycaemia OR harm* OR toxic* OR intoxic* OR complication*):ab,ti) AND ((newborn* OR new NEXT born* OR neonat* OR postnatal*):ab,ti)
